# 1-De­oxy-1-fluoro-l-galactitol

**DOI:** 10.1107/S1600536810016624

**Published:** 2010-05-12

**Authors:** Sarah F. Jenkinson, Daniel Best, Ken Izumori, Francis X. Wilson, Alexander C. Weymouth-Wilson, George W. J. Fleet, Amber L. Thompson

**Affiliations:** a Department of Organic Chemistry, Chemistry Research Laboratory, University of Oxford, Mansfield Road, Oxford OX1 3TA, England; b Rare Sugar Research Centre, Kagawa University, 2393 Miki-cho, Kita-gun, Kagawa 761-0795, Japan; c Summit PLC, 91 Milton Park, Abingdon, Oxon, OX14 4RY, England; d Dextra Laboratories Ltd, Science and Technology Centre, Whiteknights Road, Reading RG6 6BZ, England; e Department of Chemical Crystallography, Chemistry Research Laboratory, University of Oxford, Mansfield Road, Oxford OX1 3TA, England

## Abstract

The crystal structure unequivocally confirms the relative stereochemistry of the title compound, C_6_H_13_FO_5_ [6-de­oxy-6-fluoro-d-galactitol or (2*S*,3*R*,4*R*,5*S*)-6-fluoro­hexane-1,2,3,4,5-penta­ol]. The absolute stereochemistry was determined from the use of d-galactose as the starting material. In the crystal, the molecules are linked by O—H⋯O and O—H⋯F hydrogen bonds, forming a three-dimensional network with each mol­ecule acting as a donor and acceptor for five hydrogen bonds.

## Related literature

For literature regarding fluoro­galactitol and fluoro­galactose, see: Kent & Wright (1972 ▶); Jenkinson *et al.* (2010 ▶).
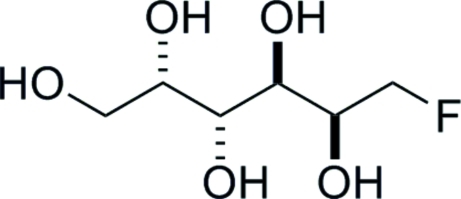


## Experimental

### 

#### Crystal data

C_6_H_13_FO_5_
*M* *_r_* = 184.16
Monoclinic, 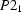

*a* = 4.7968 (3) Å
*b* = 8.5957 (5) Å
*c* = 9.8194 (7) Å
β = 103.233 (3)°
*V* = 394.12 (4) Å^3^
*Z* = 2
Mo *K*α radiation
μ = 0.15 mm^−1^
*T* = 150 K
0.40 × 0.10 × 0.05 mm

#### Data collection

Area diffractometer
Absorption correction: multi-scan (*DENZO*/*SCALEPACK*; Otwinowski & Minor, 1997 ▶) *T*_min_ = 0.88, *T*_max_ = 0.99
3069 measured reflections
947 independent reflections
788 reflections with *I* > 2σ(*I*)
*R*_int_ = 0.039

#### Refinement

*R*[*F* ^2^ > 2σ(*F* ^2^)] = 0.040
*wR*(*F* ^2^) = 0.095
*S* = 0.99
947 reflections
109 parameters
1 restraint
H-atom parameters constrained
Δρ_max_ = 0.35 e Å^−3^
Δρ_min_ = −0.36 e Å^−3^

### 

Data collection: *COLLECT* (Nonius, 2001 ▶); cell refinement: *DENZO*/*SCALEPACK* (Otwinowski & Minor, 1997 ▶); data reduction: *DENZO*/*SCALEPACK*; program(s) used to solve structure: *SIR92* (Altomare *et al.*, 1994 ▶); program(s) used to refine structure: *CRYSTALS* (Betteridge *et al.*, 2003 ▶); molecular graphics: *CAMERON* (Watkin *et al.*, 1996 ▶); software used to prepare material for publication: *CRYSTALS*.

## Supplementary Material

Crystal structure: contains datablocks global, I. DOI: 10.1107/S1600536810016624/lh5036sup1.cif

Structure factors: contains datablocks I. DOI: 10.1107/S1600536810016624/lh5036Isup2.hkl

Additional supplementary materials:  crystallographic information; 3D view; checkCIF report

## Figures and Tables

**Table 1:** Hydrogen-bond geometry (Å, °)

*D*—H⋯*A*	*D*—H	H⋯*A*	*D*⋯*A*	*D*—H⋯*A*
O4—H41⋯O8^i^	0.82	1.94	2.738 (4)	165
O10—H101⋯O12^ii^	0.82	1.95	2.730 (4)	160
O8—H81⋯O10^iii^	0.82	1.87	2.691 (4)	172
O6—H61⋯O4^iv^	0.82	1.89	2.703 (4)	170
O12—H121⋯F1^v^	0.84	2.08	2.895 (3)	163

Symmetry codes: (i) 
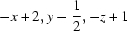
; (ii) 
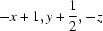
; (iii) 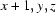; (iv) 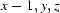; (v) 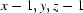.

**Fig. 1.**
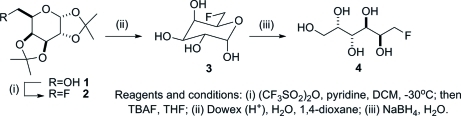
Synthetic Scheme.

**Fig. 2.**
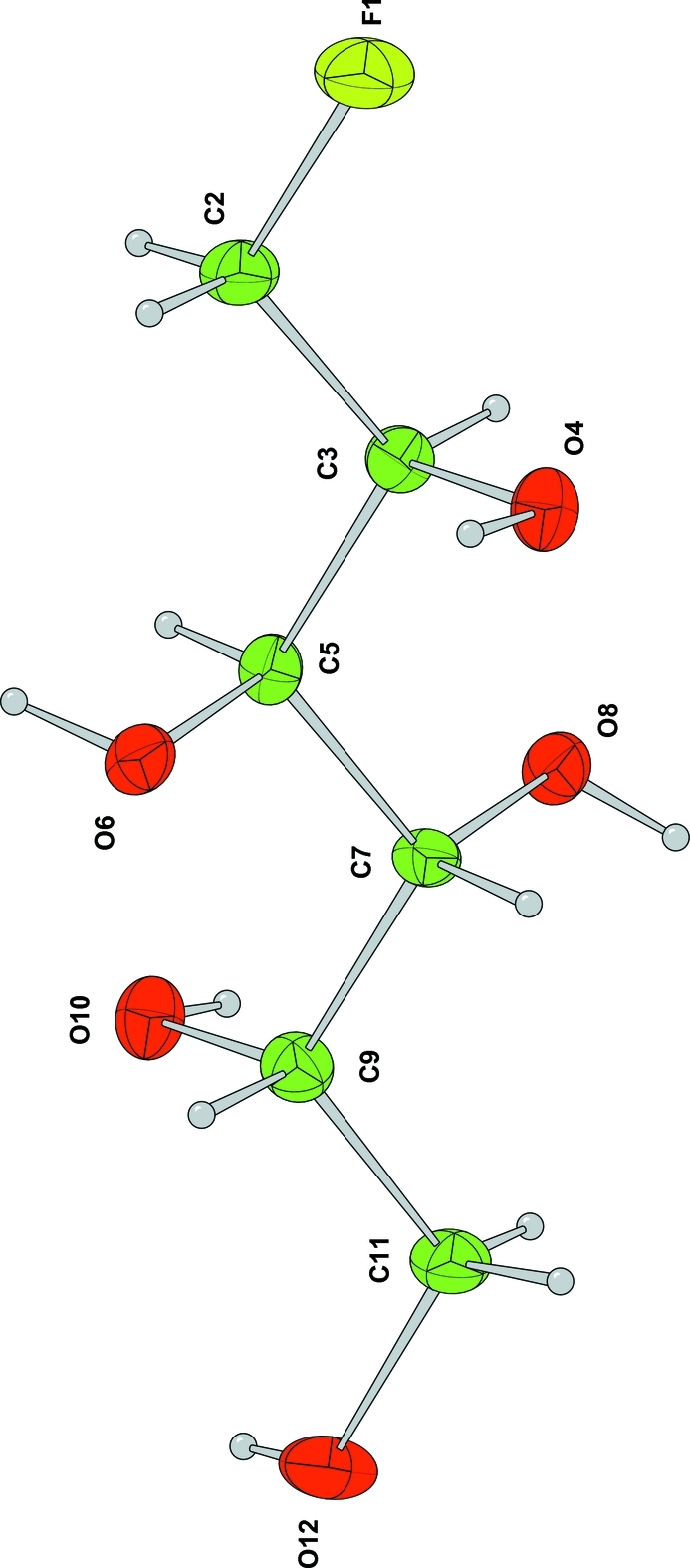
The title compound with displacement ellipsoids drawn at the 50% probability level. H atoms are shown as spheres of arbitrary radius.

**Fig. 3.**
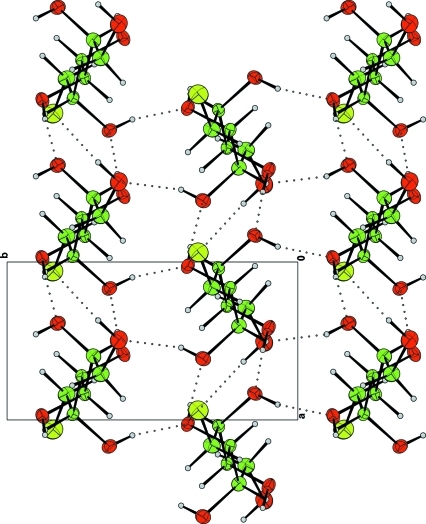
Packing diagram for the title compound projected along the c-axis. Hydrogen bonds are indicated by dotted lines.

**Fig. 4.**
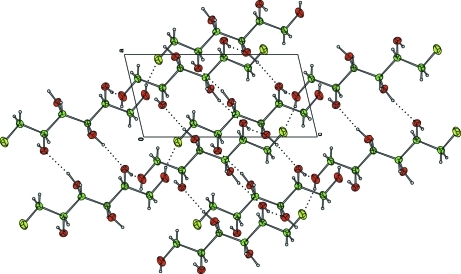
Packing diagram for the title compound projected along the b-axis. Hydrogen bonds are indicated by dotted lines.

## supplementary crystallographic information

### Comment

1-Deoxy-1-fluoro-L-galactitol [6-deoxy-6-fluoro-D-galactitol, (2*S*,3*R*,4*R*,5*S*)-6-fluorohexane-1,2,3,4,5-pentaol] 3 was prepared in 88% yield by reduction of 6-deoxy-6-fluoro-D-galactose 2, itself readily available from D-galactose (Jenkinson *et al.*, 2010) with sodium borohydride in water (see fig. 1).

1-Deoxy-1-fluoro-L-galactitol 3 (Fig. 2) exists as an extensively hydrogen bonded lattice with each molecule acting as a donor and acceptor for 5 hydrogen bonds (Fig. 3 and Fig. 4). Only classical hydrogen bonding is considered.

### Experimental

The title compound was recrystallised by vapour diffusion from a mixture of methanol and water: m.p. 445-447 K, [α]_D_^25^ +4.1 (*c* 1.06, H_2_O) {Lit. (Kent & Wright, 1972) m.p. 446-447 K, [α]_D_^21^ +4.2 (*c* 0.5, H_2_O)}.

### Refinement

In the absence of significant anomalous scattering, Friedel pairs were merged and the absolute configuration was assigned from the starting material.

The H atoms were all located in a difference map, but those attached to carbon atoms were repositioned geometrically. The H atoms were initially refined with soft restraints on the bond lengths and angles to regularize their geometry (C—H in the range 0.93–0.98, O—H = 0.82 Å) and *U*_iso_(H) (in the range 1.2–1.5 times *U*_eq_ of the parent atom), after which the positions were refined with riding constraints.

### Crystal data

**Table:**

C_6_H_13_FO_5_	*F*(000) = 196
*M**_r_* = 184.16	*D*_x_ = 1.552 Mg m^−^^3^
Monoclinic, *P*2_1_	Mo *K*α radiation, λ = 0.71073 Å
Hall symbol: P 2yb	Cell parameters from 828 reflections
*a* = 4.7968 (3) Å	θ = 5–27°
*b* = 8.5957 (5) Å	µ = 0.15 mm^−^^1^
*c* = 9.8194 (7) Å	*T* = 150 K
β = 103.233 (3)°	Needle, colourless
*V* = 394.12 (4) Å^3^	0.40 × 0.10 × 0.05 mm
*Z* = 2	

### Data collection

**Table:**

Area diffractometer	788 reflections with *I* > 2σ(*I*)
graphite	*R*_int_ = 0.039
ω scans	θ_max_ = 27.5°, θ_min_ = 5.2°
Absorption correction: multi-scan (*DENZO*/*SCALEPACK*; Otwinowski & Minor, 1997)	*h* = −6→6
*T*_min_ = 0.88, *T*_max_ = 0.99	*k* = −10→11
3069 measured reflections	*l* = −12→12
947 independent reflections	

### Refinement

**Table:**

Refinement on *F*^2^	Primary atom site location: structure-invariant direct methods
Least-squares matrix: full	Hydrogen site location: inferred from neighbouring sites
*R*[*F*^2^ > 2σ(*F*^2^)] = 0.040	H-atom parameters constrained
*wR*(*F*^2^) = 0.095	Method = Modified Sheldrick *w* = 1/[σ^2^(*F*^2^) + ( 0.04*P*)^2^ + 0.16*P*], where *P* = [max(*F*_o_^2^,0) + 2*F*_c_^2^]/3
*S* = 0.99	(Δ/σ)_max_ = 0.0001
947 reflections	Δρ_max_ = 0.35 e Å^−^^3^
109 parameters	Δρ_min_ = −0.36 e Å^−^^3^
1 restraint	

### Fractional atomic coordinates and isotropic or equivalent isotropic displacement parameters (Å^2^)

**Table:**

	*x*	*y*	*z*	*U*_iso_*/*U*_eq_	
F1	1.0608 (4)	0.8416 (3)	0.81046 (18)	0.0300	
C2	0.8382 (7)	0.7956 (4)	0.6953 (3)	0.0222	
C3	0.9617 (6)	0.7729 (3)	0.5690 (3)	0.0165	
O4	1.1674 (4)	0.6479 (3)	0.5922 (2)	0.0198	
C5	0.7225 (6)	0.7433 (3)	0.4383 (3)	0.0161	
O6	0.5806 (5)	0.6003 (2)	0.4502 (2)	0.0195	
C7	0.8349 (6)	0.7338 (3)	0.3047 (3)	0.0157	
O8	0.9746 (4)	0.8784 (3)	0.2914 (2)	0.0195	
C9	0.5959 (7)	0.7026 (4)	0.1752 (3)	0.0192	
O10	0.3879 (4)	0.8258 (3)	0.1522 (2)	0.0207	
C11	0.7088 (7)	0.6776 (4)	0.0451 (3)	0.0215	
O12	0.4930 (5)	0.6156 (3)	−0.0668 (2)	0.0275	
H21	0.6950	0.8781	0.6774	0.0260*	
H22	0.7491	0.6999	0.7173	0.0258*	
H31	1.0631	0.8691	0.5540	0.0186*	
H51	0.5826	0.8297	0.4297	0.0192*	
H71	0.9744	0.6473	0.3126	0.0169*	
H91	0.4965	0.6083	0.1918	0.0218*	
H111	0.7818	0.7757	0.0180	0.0236*	
H112	0.8650	0.6017	0.0659	0.0245*	
H41	1.0976	0.5692	0.6169	0.0297*	
H101	0.4579	0.9011	0.1221	0.0326*	
H81	1.0943	0.8692	0.2436	0.0307*	
H61	0.4434	0.6205	0.4847	0.0315*	
H121	0.3696	0.6866	−0.0851	0.0400*	

### Atomic displacement parameters (Å^2^)

**Table:**

	*U*^11^	*U*^22^	*U*^33^	*U*^12^	*U*^13^	*U*^23^
F1	0.0335 (11)	0.0350 (11)	0.0187 (9)	0.0018 (10)	0.0002 (8)	−0.0045 (8)
C2	0.0217 (15)	0.0270 (17)	0.0166 (14)	0.0023 (12)	0.0019 (12)	−0.0039 (12)
C3	0.0168 (14)	0.0140 (13)	0.0181 (13)	0.0019 (11)	0.0029 (11)	−0.0004 (11)
O4	0.0176 (11)	0.0202 (11)	0.0222 (10)	0.0029 (8)	0.0054 (8)	0.0048 (8)
C5	0.0162 (14)	0.0136 (14)	0.0194 (14)	0.0000 (11)	0.0058 (11)	0.0011 (11)
O6	0.0229 (12)	0.0169 (10)	0.0215 (10)	−0.0052 (9)	0.0109 (9)	−0.0024 (8)
C7	0.0172 (14)	0.0148 (15)	0.0149 (13)	−0.0012 (12)	0.0029 (11)	−0.0027 (11)
O8	0.0208 (12)	0.0165 (11)	0.0233 (10)	−0.0033 (9)	0.0094 (9)	−0.0024 (9)
C9	0.0200 (14)	0.0201 (15)	0.0173 (14)	−0.0015 (12)	0.0037 (12)	0.0006 (12)
O10	0.0190 (11)	0.0217 (11)	0.0223 (11)	0.0015 (9)	0.0064 (8)	0.0042 (9)
C11	0.0227 (16)	0.0257 (17)	0.0145 (14)	0.0006 (13)	0.0011 (12)	−0.0018 (13)
O12	0.0312 (14)	0.0274 (12)	0.0201 (11)	0.0025 (10)	−0.0017 (10)	−0.0078 (10)

### Geometric parameters (Å, °)

**Table:**

F1—C2	1.422 (3)	C7—O8	1.432 (4)
C2—C3	1.504 (4)	C7—C9	1.528 (4)
C2—H21	0.975	C7—H71	0.992
C2—H22	0.974	O8—H81	0.824
C3—O4	1.441 (4)	C9—O10	1.437 (4)
C3—C5	1.534 (4)	C9—C11	1.513 (4)
C3—H31	0.987	C9—H91	0.973
O4—H41	0.816	O10—H101	0.815
C5—O6	1.422 (4)	C11—O12	1.429 (3)
C5—C7	1.530 (3)	C11—H111	0.973
C5—H51	0.992	C11—H112	0.979
O6—H61	0.825	O12—H121	0.840
			
F1—C2—C3	109.0 (3)	C5—C7—C9	112.2 (2)
F1—C2—H21	108.1	O8—C7—C9	110.7 (2)
C3—C2—H21	109.9	C5—C7—H71	109.7
F1—C2—H22	110.2	O8—C7—H71	109.6
C3—C2—H22	110.4	C9—C7—H71	107.3
H21—C2—H22	109.1	C7—O8—H81	111.8
C2—C3—O4	110.5 (2)	C7—C9—O10	111.3 (2)
C2—C3—C5	110.6 (2)	C7—C9—C11	112.5 (2)
O4—C3—C5	111.3 (2)	O10—C9—C11	110.0 (2)
C2—C3—H31	108.3	C7—C9—H91	108.0
O4—C3—H31	107.7	O10—C9—H91	107.0
C5—C3—H31	108.3	C11—C9—H91	107.8
C3—O4—H41	110.6	C9—O10—H101	108.3
C3—C5—O6	110.8 (2)	C9—C11—O12	111.5 (3)
C3—C5—C7	112.5 (2)	C9—C11—H111	109.2
O6—C5—C7	107.1 (2)	O12—C11—H111	110.9
C3—C5—H51	108.0	C9—C11—H112	108.9
O6—C5—H51	109.1	O12—C11—H112	107.3
C7—C5—H51	109.3	H111—C11—H112	109.2
C5—O6—H61	107.1	C11—O12—H121	104.2
C5—C7—O8	107.3 (2)		

### Hydrogen-bond geometry (Å, °)

**Table:**

*D*—H···*A*	*D*—H	H···*A*	*D*···*A*	*D*—H···*A*
C2—H21···O6^i^	0.97	2.49	3.409 (4)	157
O4—H41···O8^ii^	0.82	1.94	2.738 (4)	165
O10—H101···O12^iii^	0.82	1.95	2.730 (4)	160
O8—H81···O10^iv^	0.82	1.87	2.691 (4)	172
O6—H61···O4^v^	0.82	1.89	2.703 (4)	170
O12—H121···F1^vi^	0.84	2.08	2.895 (3)	163

Symmetry codes: (i) −*x*+1, *y*+1/2, −*z*+1; (ii) −*x*+2, *y*−1/2, −*z*+1; (iii) −*x*+1, *y*+1/2, −*z*; (iv) *x*+1, *y*, *z*; (v) *x*−1, *y*, *z*; (vi) *x*−1, *y*, *z*−1.
